# Exploring factors associated with views on sharing of certain interim trial result measures by the data safety monitoring board (DSMB) with non-DSMB members

**DOI:** 10.1186/s13063-018-2938-3

**Published:** 2018-11-12

**Authors:** Victoria Borg Debono, Lawrence Mbuagbaw, James Paul, Norman Buckley, Lehana Thabane

**Affiliations:** 1 Health Research Methods, Evidence, and Impact, McMaster University, Hamilton, ON Canada; 2 Biostatistics Unit, St Joseph’s Healthcare, Hamilton, ON Canada; 3 Department of Anesthesia, McMaster University, Hamilton, ON Canada

**Keywords:** Data Safety Monitoring Board (DSMB), Data Monitoring Committee (DMC), Interim result sharing, Survey

## Abstract

**Background:**

Sharing interim result measures by the Data Safety Monitoring Board (DSMB) with non-DSMB members is an important issue that can affect trial integrity. Currently, it is unclear if there are demographic factors associated with sharing such information. This study’s objective is to primarily explore the demographic factors associated with the DSMB sharing certain interim result measures and secondarily, explore demographic factors associated with the perceived usefulness in sharing certain interim result measures, with non-DSMB members.

**Methods:**

We conducted an online survey of members of the Society of Clinical Trials (SCT) and International Society of Clinical Biostatistics (ISCB) in 2015 asking their professional views on the DSMB sharing interim trial results, specifically the interim control event rate (IControlER), interim combined even rate (ICombinedER), adaptive conditional power (ACP) and unconditional conditional power (UCP) with non-DSMB members. Binary logistic and multiple linear regressions were used to explore if demographic factors were associated with sharing a certain interim result measure and the perceived usefulness of sharing that interim result measure, respectively. Multiple imputation (MI) was used to evaluate the impact of missing data as a sensitivity analysis.

**Results:**

Approximately 3136 (936 from SCT + ~ 2200 from ISCB) members were invited (response rate of 12%; [371/3136]. Two main findings: (1) involvement in more than 15 private industry-sponsored trials was associated with not endorsing the sharing of the IControlER (odds ratio [OR] = 2.92; 95% confidence interval [CI]: 1.31, 6.52]; *p* = 0.012), and (2) involvement in more than 15 private industry-sponsored trials was associated positively with an increase in the perceived usefulness in sharing the ACP by 2.35 points (beta coefficient estimate = 2.35 [95% CI: 0.45, 4.05], *p* = 0.017. The findings were similar after sensitivity analyses.

**Conclusions:**

An individual involved with more than 15 trials that had some form of private industry sponsorship is a demographic factor associated with NOT sharing the IControlER by the DSMB and an increased perceived usefulness in sharing the ACP at interim. Further studies are needed to evaluate for these demographic factors given the limitations of this study related to missing data. Due to some key limitations, regarding high non-response and missing data, we caution interpreting the results as definitive, but rather look at them as a first exploratory step to find potential associations for further evaluation.

**Electronic supplementary material:**

The online version of this article (10.1186/s13063-018-2938-3) contains supplementary material, which is available to authorized users.

## Background

Data Safety Monitoring Boards (DSMBs) are in charge of the stewardship of a trial by protecting patient safety and trial integrity by reviewing accumulating interim trial results, such as safety or efficacy results when asked to, on a periodic basis, and by responding to information or trends that threaten the safety and validity of the trial by making recommendations to the trial’s steering committee (SC) or sponsor on how to proceed in light of the information [1, 2]. A trial can be negatively affected if the DSMB were to share interim trial results with non-DSMB members who are involved in the trial [1, 3, 4] as this could introduce bias. This is a grave concern for phase III trials that are typically done to provide definitive evidence on efficacy and safety outcomes [5, 6]. For this study, principal investigator(s) (PIs), study investigators, the SC, sponsors, the independent unmasked statistician, site managers, the funder(s), or patients enrolled in the trial or any other party responsible for the conduct or completion of the trial will be referred to as non-DSMB members.

Evidence in the literature suggests that DSMBs sharing potentially unmasking interim results with non-DSMB members is prevalent and may be an issue [7]. Currently, in the literature, a review indicates there are mixed views on what type of interim results to share, with whom and under what circumstances [7]. Some circumstances where the DSMB may share potentially unmasking interim results are when the DSMB makes a recommendation for early trial termination, the DSMB has concerns with the interim results given to them for a scheduled interim review, the trial’s completion as planned is endangered, the DSMB has a concern about the safety of the trial participants, and when there is a need to share interim results with a government regulator for early drug approval [7]. Other situations for sharing could be for an adaptive confirmatory trial, where interim results are needed to make decisions about planned a priori study modifications and trials with a long follow-up, where certain types of interim results may help a particular patient group or a treating physician with future treatment [7]. As part of a larger study to investigate what should be shared by the DSMB with non-DSMB members, we conducted a survey asking professionals interested or involved in trials, their views on what interim information should be shared, with whom at interim and why [8]. We found out that the interim control event rate (IControlER), adaptive conditional power (ACP), and the unconditional conditional power (UCP) should not be shared primarily because it is unmasking of interim results. The interim combined event rate (ICombinedER) is usually known by the SC or the sponsor during a trial, making it easy to determine group rate of the new intervention group if the IControlER is known. Most respondents from the survey thought the ICombinedER should be shared with the SC. Reasons indicated for sharing the ICombinedER are that the measure is not unmasking of relative effects between groups and it helps the SC monitor the trial’s progress, trial safety and the design assumptions made in the trial’s protocol. However, it was indicated that sharing the ICombinedER and for what purpose should be specified a priori and be at the DSMB’s discretion to share with non-DSMB members, especially if the IControlER is known from the literature or other sources [8]. For this article, as a second part analysis of this survey [8] using this survey’s data, the aim was to explore if there were demographic factors associated with thinking the ICombinedER, IControlER, ACP or the UCP should be shared by the DSMB with non-DSMB members and an individual’s perceived usefulness of sharing any of these four interim results measures. Evidence from this study could help us see if certain demographic groups appear to have an interest in particular interim result measures being shared and how useful they find that information, as well as promote future research to see why those groups have that interest.

## Methods

More in-depth details on survey design, methods, ethics and additional analyses that were done for this study have been previously reported and published [8] and can be referred to there. In summary, the survey was designed with 14 questions. The first six questions asked respondents the following types of questions: (A) the type of interim result measures they thought should be shared at interim by the DSMB with non-DSMB members, and if so, with whom it should be shared and why it should be shared and (B) the usefulness of sharing that information (on a scale from 0 to 10 where 0 is “Not Useful At All” and 10 is “Very Useful”). Additional file 1 provides the definitions of these four interim results measure that were also given in the survey to respondents. The first six questions had advanced/adaptive branching such that latter parts of a particular question would appear depending on how the respondent answered an earlier part of that same question [8]. The remaining questions were demographic questions asking about the respondent’s experience with trials, their self-identified primary profession by training, and work setting. None of the questions were mandatory and no incentives were offered to participate. Respondents were not able to go back and review or change previous answers before submitting.

The survey was administered online and was conducted using FluidSurveys.com. The initial version was pilot tested with 10 health research methods experts at McMaster University, Hamilton, Ontario for content validity and clarity. Nine trial experts responded to the pilot test and provided feedback on the online survey, which was used to modify and create the final version. The target group for this survey were trialists or those interested or involved in trials. The Society of Clinical Trial (SCT), with approximately 936 members around February 2015, and the International Society of Clinical Biostatistics ((ISCB), with approximately 2200 current and past members around July 2015, were asked for help in distributing our survey on our behalf to their members. Both societies agreed and helped distribute the survey. Recruitment was with an open survey link that was advertised and distributed by SCT and ISCB via email to their member mailing list. Multiple emails were sent out on our behalf to remind potential respondents of the survey to get the best response rate following Dillman’s principles [9]. Emails with a link to the survey were sent to potential respondents during the year of 2015. Specifically for SCT, an email introducing the survey to their members mailing list was sent on February 18, 2015. The first survey distribution email with a link to the survey was on February 20, 2015. The second, third, and fourth distribution/reminder/thank you emails were sent on March 13, 2015, April 23, 2015 and May 7, 2015, respectively. For ISCB, the introduction email for the survey and the first distribution email were sent as one email on August 5, 2015. The second distribution/reminder email was sent on September 5, 2015. A flow diagram showing the number of responses from SCT and ISCB after each reminder email can be found here [8]. Prior to participating and taking the survey, potential respondents were informed on the introduction page of the online survey the following: who the study investigators were, the purpose of the study, that this survey was anonymous, and that responses will not be linked to them or their identity. They were also informed that their responses would not be shared with anyone outside of the study team and that only aggregate data would be published. By clicking the next button on the introduction page and starting the survey, they were informed that they were consenting to participate in this survey. This study received Hamilton Integrated Research Ethics Board approval [10].

### Data collection and analysis

All responses and data was collected anonymously using FluidSurveys.com, which was a secure site and data was stored on password protected and encrypted key. None of the responses could be linked to an individual. WINPEPI 11.65 [11], Excel 2010® (Microsoft, Redmond, WA, USA), and IBM SPSS Statistics 24® (IBM Corp, Armonk, NY, USA) were used to analyse the results. We report results in aggregate by count and percentages, and by means and standard deviations where appropriate, with 95% confidence intervals (95% CIs). Five demographic factors were used as exploratory variables for both our binary logistic and multiple linear regressions: (1) number of trials respondent has been involved (≤ 15 trials [coded as 0, reference category] **or** > 15 trials [coded as 1), (2) number of trials the respondent has been involved with that had a DSMB monitoring the trial (≤ 15 trials [coded as 0, reference category] **or** > 15 trials [coded as 1]), (3) number of trials the respondent has been involved with that had some form of private industry sponsorship (≤ 15 trials [coded as 0, reference category] **or** > 15 trials [coded as 1]), (4) primary profession by training (mathematician/statistician/biostatistician **or **methodological scientist/research methodologist **or** other (reference category)) and (5) usual work setting of respondents (university or academic institution **or** private **or** contracted research company **or** other (reference category)). These groupings were determined so as to satisfy the general rule of thumb of 10 events per degree of freedom [12] for creating stable models using logistic regression. With an estimated standard population size of 10,000, a confidence level of 95%, an expected response distribution frequency of 50%, and a margin of error of 5%, the total sample required was calculated to be 370 [13]. For the primary objective and analysis, four binary logistic regressions were done for sharing each of the interim result measures by the DSMB with non-DSMB members (outcome: Yes or No for sharing, the ICombinedER, IControlER, ACP and UCP). A Hosmer-Lemeshow test was done to evaluate the goodness-of-fit of the binary logistic regressions. A second set of four binary logistic regressions was done using an imputed dataset as a sensitivity analysis to evaluate for the impact of missing data in the original dataset.

For the secondary objective and analysis, four multiple linear regressions were done for the individual’s perceived usefulness of sharing each of the four interim result measures by the DSMB with non-DSMB members (outcome: on a scale from 0 – Not Useful At All to 10 – Very Useful, treated as a continuous outcome, for the IControER, ICombinedER, ACP, and UCP). Respondents regarding their thoughts on the usefulness of sharing each of the interim results would have first answered in the survey that a particular interim result measure (e.g. IControlER) should be shared with a non-DSMB member(s) before answering the question on their perceived usefulness of having that information shared at interim. Assumptions associated with linear regression were assessed for by testing for linearity, normality, homoscedasticity, multicollinearity, and independence. Bootstrapping with bias corrected and accelerated CIs was used for the analysis to ensure robustness of 95% CIs and significance values, which protects against any violations of the assumptions for normality or homoscedasticity. A second set of four multiple linear regressions was done using an imputed dataset as a sensitivity analysis to evaluate for the impact of missing data in the original dataset. All demographic factors were entered into the regression simultaneously using a multivariable approach for both the logistic and multiple linear regressions. The regression analyses were used to explore potential associations, not build models for prediction as this is a cross-sectional survey. Multiple imputation (MI), using the full conditional specification method that implements the Markov Chain Monte Carlo (MCMC) algorithm, was used for data imputation using IBM SPSS Statistics 24® [14]. The linear and binary logistic regressions and the sensitivity analyses were also done using IBM SPSS Statistics 24®.

## Results

Three-hundred and seventy-one responses (202 complete responses, 169 partial or incomplete responses) were received; response rate of 12% (371/3136). Three reminder emails were sent to SCT members by SCT and one reminder email was sent to ISCB members by ISCB, as was allowed by the organisations.

### Demographics

Table 1 summarizes the five demographic factors of the respondents. About 35.3% of the respondents have been involved in more than 15 trials and 42.0% of the respondents identified being a mathematician/statistician/biostatistician as their primary profession by training.

**Table 1:** Summary of demographic factors

**Number of trials in which respondent has been involved** ^**a**^	
Number of trials	n (%)
≤ 15 trials	72 (19.4)
> 15 trials	131 (35.3)
Unknown^A^	168 (45.3)
**Number of trials the respondent has been involved with that had a DSMB monitoring the trial** ^**b**^	
Number of trials	n (%)
≤ 15 trials	109 (29.4)
> 15 trials	88 (23.7)
Unknown^B^	174 (46.9)
**Number of trials the respondent has been involved with that had some form of private industry sponsorship** ^**c**^	
Number of trials	n (%)
≤ 15 trials	137 (36.9)
> 15 trials	64 (17.3)
Unknown^C^	170 (45.8)
**Primary profession by training** ^**d**^	
Main profession	n (%)
Mathematician/statistician/biostatistician	156 (42.0)
Methodological scientist/research methodologist	21 (5.7)
Other	26 (7.0)
Unknown^D^	168 (45.3)
**Usual work setting of respondents** ^**e**^	
Place of work	n (%)
University or academic institution	123 (33.2)
Private or contracted research company	28 (7.5)
Other	51 (13.7)
Unknown^E^	169 (46.0)

^a^Total of 203 responses to this question, percentages are based on a total of 371 respondents to the survey. ^A^Unknown because 168 respondents did not answer this question

^b^Total of 197 responses to this question, percentages are based on a total of 371 respondents to the survey. ^B^Unknown because 174 respondents did not answer this question

^c^Total of 201 responses to this question, percentages are based on a total of 371 respondents to the survey. ^C^Unknown because 170 respondents did not answer this question

^d^Total of 203 responses to this question, percentages are based on a total of 371 respondents to the survey. ^D^Unknown because 168 respondents did not answer this question

^e^Total of 202 responses to this question. ^E^Unknown because 169 respondents did not answer this question

### Responses about sharing the IControlER, ICombinedER, ACP, and UCP and perceived usefulness in sharing

Table 2 shows the results based on the responses regarding what interim result measure should be shared by the DSMB of a trial with non-DSMB members and their perceived usefulness of sharing a certain interim result measures if they indicated that interim result measures should be shared. The only interim result measure the majority of respondents thought should be shared with non-DSMB members by the DSMB was the ICombinedER (168/262; 64.1% [95% CI: 58.0, 69.9]). For those 168 respondents that thought the ICombinedER should be shared, 146 answered the question regarding the usefulness of sharing that information with non-DSMB members with a mean of 6.97 [95% CI: 6.62, 7.31]. For all other interim measures, the majority of respondents thought it should not be shared (see Table 2 for more details).

**Table 2:** Summary of respondents’ thoughts on sharing the IControlER, ICombinedER, ACP, and UCP and its usefulness

**Interim combined event rate**	
1. During an ongoing randomized controlled trial (RCT), do you think that the Data Safety Monitoring Board (DSMB) for an RCT should share the interim combined event rate with ANY of the following parties?	
Response	Results [n/N; % (95% CI)]
Yes^A^	168/262; 64.1% (58.0% to 69.9%)
No^B^	94/262; 35.9% (30.1% to 41.7%)
2. How useful is it to share the interim combined event rates at interim? (On a scale from 0 to 10 where 0 is Not Useful at All and 10 is Very Useful) *(This question was answered only by those who were categorized as “Yes” to 1. (above))*	
Number of responses to question	Results (mean (95% CI); median [IQR])
146	6.97 (6.62 to 7.31); 7 [6–8]
**Interim control event rate**	
3. During an ongoing randomized controlled (RCT), do you think that the Data Safety Monitoring Board (DSMB) for an RCT should share the interim control event rate with ANY of the following parties?	
Response	Results [n/N; % (95% CI)]
Yes^A^	88/237; 37.1% (31.0% to 43.3%)
No^B^	149/237; 62.9% (56.7% to 69.0%)
4. How useful is it to share the interim control event rates at interim? (On a scale from 0 to 10 where 0 is Not Useful at All and 10 is Very Useful) *(This question was answered only by those who were categorized as “Yes” to 3. (above))*	
Number of responses to question	Results (mean (95% CI); median [IQR])
72	7.03 (6.55 to 7.50); 7 [5–8]
**Adaptive conditional power**	
5. During an ongoing randomized controlled trial (RCT), do you think that the Data Safety Monitoring Board (DSMB) for an RCT should share the adaptive conditional power with ANY of the following parties?	
Response	Results [n/N; % (95% CI)]
Yes^A^	80/224; 35.7% (29.4% to 42.0%)
No^B^	144/224; 64.3% (58.0% to 70.6%)
6. How useful is it to share the adaptive conditional power at interim? (On a scale from 0 to 10 where 0 is Not Useful at All and 10 is Very Useful) *(This question was answered only by those who were categorized as “Yes” to 5. (above))*	
Number of responses to question	Results (mean (95% CI); median [IQR])
66	6.64 (6.08 to 7.20); 7 [5–8]
**Unconditional conditional power**	
7. During an ongoing randomized controlled trial (RCT), do you think that the Data Safety Monitoring Board (DSMB) for an RCT should share the unconditional conditional power with ANY of the following parties?	
Response	Results [n/N; % (95% CI)]
Yes^A^	82/208; 39.4% (32.8% to 46.1%)
No^B^	126/208; 60.6% (53.9% to 67.2%)
8. How useful is it to share the unconditional conditional power at interim? (On a scale from 0 to 10 where 0 is Not Useful at All and 10 is Very Useful) *This question was answered only by those who were categorized as “Yes” to 7. (above))*	
Number of responses to question	Results (mean (95% CI); median [IQR])
67	6.64 (6.08 to 7.20); 7 [5–8]

^A^Respondent had to select any of the following parties for a Yes categorization: A. The Sponsor, B. The Steering Committee, C. The Investigator(s), D. The Funder(s), or E. Other, Please Specify)

^B^Respondent had to select F. None of the Above for a No categorization

### Demographic factors associated with sharing the IControlER, ICombinedER, ACP, and UCP

Table 3 summarizes the results of the binary logistic regression of potential demographic factors associated with sharing ICombinedER, IControlER, ACP, and UCP for our primary analysis before and after the sensitivity analysis. One demographic factor before the sensitivity analysis, the primary profession by training, was significantly associated with not sharing the ICombinedER [coded as No, do not share the ICombinedER (0), Yes, share the ICombinedER (1)], with an odds ratio of 0.25 [95% CI: 0.07, 0.89]; *p* = 0.019, for being a mathematician/statistician/biostatistician and an odds ratio 0.50 [95% CI: 0.10, 2.43]; *p* = 0.357, for being a methodological scientist/research methodologist when compared to other (reference category). However, this finding was not corroborated with the sensitivity analysis suggestive that this analysis was sensitive to missing data.

**Table 3:** Binary logistic regressions of variables associated with sharing, ICombinedER, IControlER, ACP, or UCP with non-DSMB members by the DSMB

**ICombinedER** ^**A**^ **Hosmer-Lemeshow Test (χ** ^**2**^ ** = 1.94, D.F = 6, ** ***p*** ** = 0.93);** **[Coded as No, do not share the ICombinedER (0), Yes, share the ICombinedER (1)]** ***n*** ** = 195**		**MI sensitivity analysis for ICombinedER** ^**B**^ ***n*** ** = 264**
	**Odds ratio (95% CI);** ***p*** **value**	**Odds ratio (95% CI);** ***p*** **value**
The respondent has been involved with more than 15 trials	0.77 (0.31, 1.89); 0.558	0.74 (0.31, 1.75); 0.484
The respondent has been involved with more than 15 trials that had a DSMB monitoring the trial	0.82 (0.36, 1.84); 0.632	0.73 (0.35, 1.55); 0.414
The respondent has been involved with more than 15 trials that had some form of private industry sponsorship	1.29 (0.61, 2.76); 0.534	1.32 (0.56, 3.10); 0.514
Primary profession by training		
● Other (reference category)	1	1
● Mathematician/statistician/biostatistician	0.25 (0.07, 0.89); 0.019	0.40 (0.13, 1.29); 0.123
● Methodological scientist/research Methodologist	0.50 (0.10, 2.43); 0.357	0.84 (0.20, 3.57); 0.813
Usual work setting of respondents		
● Other (reference category)	1	1
● University or academic institution	0.94 (0.44, 2.02); 0.869	0.96 (0.49, 1.85); 0.892
● Private/contracted research company	1.11 (0.39, 3.17); 0.836	1.13 (0.38, 3.37); 0.820
**IControlER** ^**A**^ **Hosmer-Lemeshow test (χ** ^**2**^ ** = 4.526, D.F = 8, ** ***p*** ** = .807);** **[Coded as Yes, share the IControlER (0), No, do not share the IControlER (1)]** ***n*** ** = 195**		**MI sensitivity analysis for IControlER** ^**B**^ ***n*** ** = 264**
	Odds ratio (95% CI); *p* value	Odds ratio (95% CI); *p* value
The respondent has been involved with more than 15 trials	0.87 (0.36, 2.12); 0.774	0.78 (0.34, 1.80); 0.551
The respondent has been involved with more than 15 trials that had a DSMB monitoring the trial	0.82 (0.35, 1.90); 0.674	0.94 (0.40, 2.19); 0.875
The respondent has been involved with more than 15 trials that had some form of private industry sponsorship	2.92 (1.31, 6.52); 0.012	2.79 (1.11, 7.00); 0.031
Primary profession by training		
● Other (reference category)	1	1
● Mathematician/statistician/biostatistician	0.70 (0.26, 1.88); 0.505	0.68 (0.26, 1.80); 0.437
● Methodological scientist/research Methodologist	0.38 (0.11, 1.34); 0.154	0.42 (0.13, 1.36); 0.146
Usual work setting of respondents		
● Other (reference category)	1	1
● University or academic institution	0.10 (0.47, 2.12); 0.993	0.99 (0.49, 1.99); 0.977
● Private/contracted research company	1.06 (0.36, 3.10); 0.900	1.28 (0.39, 4.19); 0.672
**ACP** ^**A**^ **Hosmer-Lemeshow test (χ**^**2**^ **= 2.39, D.F = 7, *p* = 0 .94);** **[Coded as Yes, share the ACP (0), No, do not share the ACP (1)]** ***n*** ** = 196**		**MI sensitivity analysis for ACP** ^**B**^ ***n*** ** = 264**
	Odds ratio (95% CI); *p* value	Odds ratio (95% CI); *p* value
The respondent has been involved with more than 15 trials	1.08 (0.45, 2.59); 0.868	1.06 (0.46, 2.45); 0.883
The respondent has been involved with more than 15 trials that had a DSMB monitoring the trial	1.73 (0.75, 4.00); 0.237	1.75 (0.48, 6.41); 0.362
The respondent has been involved with more than 15 trials that had some form of private industry sponsorship	1.48 (0.66, 3.32); 0.358	1.41 (0.58, 3.46); 0.438
Primary profession by training		
● Other (reference category)	1	1
● Mathematician/statistician/biostatistician	0.59 (0.22, 1.62); 0.305	0.67 (0.27, 1.65); 0.375
● Methodological scientist/research Methodologist	0.85 (0.23, 3.24); 0.830	0.85 (0.19, 3.69); 0.819
Usual work setting of respondents		
● Other (reference category)	1	1
● University or academic institution	1.19 (0.55, 2.60); 0.694	0.93 (0.43, 1.98); 0.838
● Private/contracted research company	0.50 (0.18, 1.39); 0.189	0.51 (0.19, 1.39); 0.186
**UCP** ^**A**^ **Hosmer-Lemeshow test (χ**^**2**^ **= 2.664, D.F = 7,** ***p* = .914);** **[Coded as Yes, share the UCP (0), No, do not share UCP (1)]** ***n*** ** = 192**		**MI sensitivity analysis for UCP** ^**B**^ ***n*** ** = 264**
	Odds ratio (95% CI); *p* value	Odds ratio (95% CI); *p* value
The respondent has been involved with more than 15 trials	1.65 (0.69, 3.92); 0.266	1.51 (0.63, 3.63); 0.350
The respondent has been involved with more than 15 trials that had a DSMB monitoring the trial	1.04 (0.46, 2.35); 0.937	1.01 (0.31, 3.32); 0.987
The respondent has been involved with more than 15 trials that had some form of private industry sponsorship	1.11 (0.52, 2.39); 0.798	1.06 (0.53, 2.15); 0.863
Primary profession by training		
● Other (reference category)	1	1
● Mathematician/statistician/biostatistician	0.60 (0.22, 1.62); 0.341	0.66 (0.29, 1.51); 0.326
● Methodological scientist/research Methodologist	0.28 (0.08, 0.99); 0.052	0.26 (0.074, 0.94); 0.039
Usual work setting of respondents		
● Other (reference category)	1	1
● University or academic institution	1.28 (0.60, 2.71); 0.526	1.00 (0.43, 2.31); 0.995
● Private/contracted research company	0.67 (0.25, 1.79); 0.416	0.68 (0.22, 2.09); 0.481

*ACP* adaptive conditional power, *D.F*. degrees of freedom, *CI* confidence interval, *DSMB* Data Safety Monitoring Board, *ICombinedER* interim combined event rate, *IControlER* interim control event rate, *UCP* unconditional conditional power, *MI* multiple imputation

^A^CIs and standard errors based on 1000 bootstrap samples with 95% bias corrected and accelerated CIs reported

^B^Results based on pooled results from sensitivity analysis with multiple imputation. The Hosmer-Lemeshow test is not provided for pooled results analysis

The respondent having been involved with more than 15 trials that had some form of private industry sponsorship was significantly associated with not sharing the IControlER [coded as Yes, share the IControlER (0), No, do not share the IControlER (1)], with an odds ratio of 2.92 [95% CI: 1.31, 6.52]; *p* = 0.012, when compared to baseline. This finding was corroborated with the sensitivity analysis suggestive that this analysis was not sensitive to missing data.

None of the demographic factors were significantly associated with sharing the ACP both before and after the sensitivity analysis suggestive that this analysis was not sensitive to missing data.

One variable with the sensitivity analysis, the primary profession by training, was significantly associated with sharing the UCP [coded as Yes, share the UCP (0), No, do not share UCP (1)], with an odds ratio of 0.66 [95% CI: 0.29, 1.51]; *p* = 0.326 for being a mathematician/statistician/biostatistician and odds ratio of 0.26 [95% CI: 0.074, 0.94]; *p* = 0.039 for being a methodological scientist/research methodologist when compared to Other (reference category). This association was not shown before doing the MI sensitivity analysis, suggestive that this analysis was sensitive to and impacted by missing data.

### Demographic factors associated with the perceived usefulness in sharing the IControlER, ICombinedER, ACP, and UCP

Table 4 summarizes the results of the linear regression of potential factors associated with the perceived usefulness with sharing ICombinedER, IControlER, ACP, and UCP for our secondary analysis. A respondent having been involved with more than 15 trials that had some form of private industry sponsorship was the only significant demographic factor [*b* = 2.35 (95% CI: 0.45, 4.05), *p* = 0.017] associated with the perceived usefulness with sharing the ACP with non-DSMB member by the DSMB. Therefore, for this particular factor, which was dichotomized, a person having been involved with more than 15 trials that had some form of private sponsorship would suggest an increase of 2.35 points in regard to their perceived usefulness in sharing the ACP on a scale from 0 to 10 where 0 is “Not Useful At All” and 10 is “Very Useful”. This finding and the direction of the effect was corroborated with the sensitivity analysis suggesting that this analysis was not sensitive to missing data.

**Table 4:** Multiple linear regressions of variables associated with the perceived usefulness with sharing the ICombinedER, IControlER, ACP, or UCP

	Estimated coefficient (95% CI); *p* value	Sensitivity analysis^D^ Estimated coefficient (95% CI); *p* value
**ICombinedER** ^**A**^ ***n*** = **112 (R**^**2**^** = 0.11,** ***p* = 0.11)**		***n*** ** = 163**
The respondent has been involved with more than 15 trials	0.11 (−1.37, 1.42); 0.860	0.22 (− 1.25, 1.70); 0.746
The respondent has been involved with more than 15 trials that had a DSMB monitoring the trial	0.69 (− 0.29, 1.67); 0.230	0.69 (− 0.46, 1.83); 0.224
The respondent has been involved with more than 15 trials that had some form of private industry sponsorship	−0.35 (− 1.42, 0.77); 0.501	− 0.28 (− 1.19, 0.63); 0.547
Primary profession by training		
● Other (reference category)		
● Mathematician/statistician/biostatistician	0.87 (−0.17, 2.06); 0.127	0.37 (−0.64, 1.38); 0.472
● Methodological scientist/research Methodologist	1.03 (−0.47, 2.47); 0.159	0.86 (−0.48, 2.21); 0.208
Usual work setting of respondents		
● Other (reference category)		
● University or academic institution	−0.70 (− 1.83, 0.45); 0.271	−0.59 (− 1.76, 0.60); 0.317
● Private/contracted research company	−1.03 (− 2.40, 0.40); 0.139	− 0.74 (− 2.68, 1.20); 0.417
**IControlER** ^**B**^ ***n*** ** = 59 (R** ^**2**^ ** = 0.092,** ***p*** ** = 0.64)**		***n*** ** = 85**
The respondent has been involved with more than 15 trials	0.88 (− 0.87, 2.60); 0.282	0.86 (− 0.81, 2.52); 0.301
The respondent has been involved with more than 15 trials that had a DSMB monitoring the trial	− 0.75 (− 2.44, 0.84); 0.356	−0.87 (− 2.25, 0.51); 0.214
The respondent has been involved with more than 15 trials that had some form of private industry sponsorship	0.62 (− 0.89, 1.78); 0.441	0.73 (− 0.93, 2.40); 0.372
Primary profession by training		
● Other (reference category)		
● Mathematician/statistician/biostatistician	1.44 (−1.88, 5.67); 0.329	1.05 (−0.57, 2.67); 0.202
● Methodological scientist/research methodologist	0.96 (−1.91, 4.64); 0.534	0.45 (− 1.45, 2.34); 0.641
Usual work setting of respondents		
● Other (reference category)		
● University or academic institution	0.27 (−1.72, 2.09); 0.762	0.14 (−0.98, 1.26); 0.807
● Private/contracted research company	0.17 (−1.89, 2.61); 0.871	0.086 (− 1.59, 1.76); 0.919
**ACP** ^**C**^ ***n* = 51 (R**^**2**^ **= 0.23,** ***p* = 0.11)**		***n*** ** = 77**
The respondent has been involved with more than 15 trials	−0.68 (− 2.18, 0.67); 0.411	−0.58 (− 1.99, 0.83); 0.410
The respondent has been involved with more than 15 trials that had a DSMB monitoring the trial	− 0.53 (− 2.39, 1.35); 0.515	− 0.27 (− 1.48, 0.94); 0.665
The respondent has been involved with more than 15 trials that had some form of private industry sponsorship	2.35 (0.45, 4.05); 0.017	1.96 (0.48, 3.44); 0.011
Primary profession by training		
● Other (reference category)		
● Mathematician/statistician/biostatistician	0.52 (−1.35, 2.99); 0.614	−0.096 (− 1.54, 1.35); 0.896
● Methodological scientist/research methodologist	1.64 (−0.80, 5.10); 0.279	0.78 (− 1.36, 2.92); 0.466
Usual work setting of respondents		
● Other (reference category)		
● University or academic institution	1.21 (−1.07, 4.32); 0.335	0.96 (− 0.78, 2.69); 0.259
● Private/contracted research company	0.44 (− 2.07, 3.58); 0.779	0.235 (−1.84, 2.31); 0.815
**UCP** ^**B**^ ***n* = 60 (R**^**2**^ **= 0.051,** ***p* = 0.90)**		***n*** ** = 82**
The respondent has been involved with more than 15 trials	0.10 (−1.78, 2.02); 0.906	0.07 (− 1.48, 1.61); 0.933
The respondent has been involved with more than 15 trials that had a DSMB monitoring the trial	−0.32 (− 2.10, 1.48); 0.738	−0.068 (− 1.64, 1.51); 0.932
The respondent has been involved with more than 15 trials that had some form of private industry sponsorship	0.22 (− 1.58, 1.88); 0.780	0.063 (− 1.38, 1.51); 0.932
Primary profession by training		
● Other (reference category)		
● Mathematician/statistician/biostatistician	0.060 (−2.28, 2.29); 0.955	−0.55 (− 2.50, 1.39); 0.575
● Methodological scientist/research methodologist	1.45 (−1.04, 4.26); 0.256	0.86 (−1.67, 3.40); 0.497
Usual work setting of respondents		
● Other (reference category)		
● University or academic institution	1.00 (−1.21,,3.20); 0.405	1.04 (−0.45, 2.53); 0.169
● Private/contracted research company	1.02 (−2.06, 4.34); 0.508	1.15 (−0.89, 3.20); 0.265

*ACP* adaptive conditional power, *DSMB* Data Safety Monitoring Board, *CI* confidence interval, *ICombinedER* interim combined event rate, *IControlER* interim control event rate, *UCP* unconditional conditional power

^A^CIs and standard errors based on 1000 bootstrap samples with 95% bias corrected and accelerated CIs reported in brackets

^B^CIs and standard errors based on 997 bootstrap samples with 95% bias corrected and accelerated CIs reported in brackets

^C^CIs and standard errors based on 995 bootstrap samples with 95% bias corrected and accelerated CIs reported in brackets

^D^Results based on pooled results from sensitivity analysis with multiple imputation

None of the other variables were associated with the perceived usefulness in sharing the ICombinedER, IControlER, ACP, or UCP with non-DSMB members by the DSMB, which was also corroborated with the sensitivity analysis.

## Discussion

### Key findings

Our results empirically showed that there may be some key demographic factors of trialists or those interested in trials that are associated with sharing certain pieces of interim results with non-DSMB members by the DSMB. A respondent having been involved with more than 15 trials that had some form of private industry sponsorship was significantly associated with not sharing the IControlER. The relationship with the outcome of interest was a positive one for this demographic factor since we coded the outcome for this analysis as 0 for sharing the IControl and 1 for not sharing the IControlER. So, if an individual had been involved with more than 15 trials that has some form of private industry sponsorship, their odds for endorsing NOT to share the IControlER would be 2.92 higher when compared to baseline (which was being involved with ≤ 15 trials that had some form of private industry sponsorship). This finding was not sensitive to missing data as it was corroborated by the sensitivity analysis done suggesting that more confidence can be had with this association. It is possible those with more experience with trials with private industry sponsorship (i.e. > 15 trials), understand that the IControlER can be directly unmasking of interim effects between treatment groups because in many cases, the SC or the sponsor have access to interim pooled data [8]. This was a concept found when analysing responses to this survey [8]. Thus, being given the IControlER in this case would allow them to back calculate the event rate for the intervention group. Those with more trial experience with private industry sponsorship may have had more time to come across and be introduced to this idea than those with less experience with trials that had private industry sponsorship.

The primary profession by training of an individual (mathematician/statistician/biostatistician, or methodological scientist/research methodologist or other, which could have been a combination of other professionals involved in trials, e.g. physician, epidemiologist, analyst etc.) was significantly associated with not sharing the ICombinedER. The relationship with the outcome of interest was a negative one for this demographic factor since we coded the outcome for this analysis as 0 for not sharing the ICombinedER and 1 for sharing the ICombinedER. So, if an individual was of a statistics- or mathematical-oriented profession, their odds of endorsing the sharing of the ICombinedER with non-DSMB members would be 0.25 times lowers than the reference (Other category). Additionally, if an individual was a research methodologist, their odds of endorsing the sharing of the ICombinedER with non-DSMB member by the DSMB would be 0.50 times lower than the reference (Other category). However, this finding was sensitive to the missing data we had for this analysis as the sensitivity analysis did not corroborate this result and did not find any demographic factors significant. Caution should thus be exercised when interpreting the validity of this demographic factor’s association with endorsing the sharing of the ICombinedER and will require more validation.

None of the demographic factors were significantly associated with endorsing the sharing of the ACP and this result remained robust after performing some sensitivity analysis. This does not mean that there are not any factors associated with sharing this interim result measure; it is just that we may not have captured the demographic factor with the survey.

The primary profession by training, with the sensitivity analysis, was significantly associated with endorsing the sharing of the UCP. The relationship with the outcome of interest was a negative one for this demographic factor since we coded the outcome for this analysis as 0 for sharing the UCP and 1 for not sharing the UCP. So if an individual was of a statistics- or mathematical-oriented profession, their odds of NOT endorsing the sharing of the UCP with non-DSMB members would be would be 0.66 times lower than baseline (Other). Additionally, if an individual was a methodological scientist/research methodologist, their odds of NOT endorsing the sharing of the UPC would be 0.26 times lower than baseline (Other). This finding was found in only in the sensitivity analysis and none of the demographic variables showed to be significant when the regression was done with the original data. This demonstrates that this analysis was sensitive to missing data. Caution should thus be exercised when interpreting the validity of this demographic factor’s association with endorsing the sharing of the UCP and will require more validation.

Our secondary analysis demonstrated once again that a respondent having been involved with > 15 trials that had some form of private industry sponsorship had a significant association; in this case it was significantly associated with the perceived usefulness with sharing the ACP by the DSMB with non-DSMB members. The relationship was a positive one for this demographic factor where a person having been involved with > 15 trials that had some form of private sponsorship would suggest an increase of 2.35 points in the perceived usefulness in sharing the ACP. This finding was not sensitive to missing data as it was corroborated by the sensitivity analysis done suggesting that more confidence can be had with this association. It is possible those with more experience with trials with private industry sponsorship (i.e. > 15 trials), understand a concept that was found when analysing responses to our survey, that the ACP is extremely informative of the presence or absence of a relative treatment effect between groups within a trial and hence it is partially unmasking to those responsible for the conduct of the trial.

### Findings compared to similar studies

This study is unique in that it empirically evaluates key demographic factors of those involved with trials and whether these factors have any association with sharing four main forms of interim results (ICombinedER, IControlER, ACP, and UCP) and the perceived usefulness in sharing these interim results with non-DSMB members by the DSMB. This study is an extension to a survey analysis we did that evaluated whether these same four main forms of interim result measures should be shared at interim, with whom, the perceived usefulness of sharing that result measure, and why it should be shared, by soliciting the views of those involved or interested in trials [8]. In that survey analysis, as part of a larger study to investigate what should be shared by the DSMB with non-DSMB members, we found evidence suggesting that the IControlER, the ACP, and the UCP should not be shared with non-DSMB members by the DSMB and that the ICombinedER could be shared when planned to do so in a priori manner as indicated in the protocol or the DSMB charter [8]. However, the DSMBs should use discretion when sharing the ICombinedER if the IControlER is well known in the literature or from another source as then it is unmasking of the event rate in the other group. A scenario-based survey we also did asked trial experts how they interpreted the ICombinedER, ACP, and UCP when shared in a hypothetical trial scenario [15]. We concluded from that survey that knowledge of these three interim results should not be shared by DSMB with non-DSMB members at interim as they may mislead or unmask interim results, potentially introducing trial bias [15].

Six other surveys dating from 1999 to 2011 [16–19] did not specifically focus on the issue of the DSMB sharing interim results, and were very limited in regards to the amount of evidence collected regarding what information should be shared by the DSMB, with whom and for what reason. None of these surveys did regression analyses to see if demographic factors were associated with sharing the four main forms of interim result measures we have looked at in this study or the perceived usefulness in sharing those interim result measures.

### Key Limitations

One limitation with our study is that we had a low response rate for our survey despite strong efforts to solicit and gather responses. Thus, we do not have a definitive way of knowing how our non-respondents were different from those who responded to our survey. However, we do know that the largest proportion of our respondents regarding their primary profession by training self-identified as a mathematician/statistician/biostatistician and about 54% of our respondents had experience with at least one trial. This information gathered was reassuring as many of our respondents were likely aware of what interim trial result measures were.

Additionally, our survey had a lot of missing data. We received 371 responses where 202 were complete responses, meaning those 202 respondents filled all the questions to our survey. However, 169 were partially complete or incomplete responses meaning that questions were either skipped or left blank. Particularly with our demographic information, we had 40% or more missing information from respondents. Answers from questions regarding how respondents viewed sharing four main forms of interim result measures had less missing data, most likely because these were questions asked first in the survey. We can only speculate that some of the missing data, particularly at the beginning of the survey, may have been because respondents did not know if certain interim result measures should be shared with non-DSMB members and left those questions blank. However, there was an “Other. Please specify” option for those questions with a blank space where a respondent could have written “I don’t know” and that response would have been valid. Still, this does not take away from the fact that those respondents with less experience with certain interim result measures or who simply did not have an answer made an educated guess about whether a measure should be shared.

To compensate for the missing data in this study, we did a sensitivity analysis using MI, which is the most robust form of imputation for missing data including survey data [20–22]. We compared the regression results with the dataset we originally had versus the dataset with MI to see if the analysis was sensitivity to data that was missing. If the results of the same analysis were different with the dataset we originally had versus the dataset with imputation, we exercised caution with interpreting the results. We also used bootstrapping with bias corrected and accelerated CIs for our regressions with the dataset we originally had to ensure robustness of 95% CIs and significance values found.

Moreover, as mentioned in our previous article on the initial findings from this survey [8], it is possible that a repondent could have completed the survey twice as recruitment was with an open survey link that was advertised and distributed by SCT and ISCB via email to their member mailing list. Thus, we could not provide an exclusive and identifiable link to each unique respondent. Respondent’s anonymity and privacy was a priority and we did not collect identifiable information that would allow us to reveal who filled out the survey more than once. However, if an individual was a member of both societies, it is possible that they remembered filling out the survey and would not fill it out again as the same survey was used. The survey title/introduction page would have been recognisable before clicking the start button for the survey. Due to these limitations highlighted above, we explicitly encourage caution in interpreting these as definitive results, but rather look at them as a first exploratory step to find potential associations for later study.

### Implications for Practice

Two robust analyses and findings were generated from this study as they were corroborated in the sensitive analysis; (1) an individual that has been involved with more than 15 trials that had some form of private industry sponsorship demonstrated a significant positive association for NOT sharing the IControlER, and (2) an individual that has been involved with > 15 trials that had some form of private industry sponsorship demonstrated a significant positive association with the perceived usefulness with sharing the ACP by the DSMB with non-DSMB members. The commonality between the two findings is that an individual who has been involved with more than 15 trials that had some form of private industry sponsorship is the significant demographic factor. This finding warrants more investigation into this subgroup of trialists who have experience with trials with private industry sponsorship. Hypothetically speaking, we could ask the following question based on this finding: Does having more experience with private industry sponsored trials provide trialists with a better understanding about the amount of information the IControlER and ACP provides about treatment group effects and relative group effects respectively, at interim? More knowledge of their experience can provide insight into good interim trial management practices, especially if this subgroup is already doing something preventatively to protect from trial bias.

## Conclusions

From this survey, we have done several regression analyses that have provided empirical evidence to potentially indicate that the more trials an individual has been involved with (> 15 trials) that had some form of private industry sponsorship is a potential factor associated with NOT sharing the IControlER and the perceived usefulness with sharing the ACP. This demographic factor should be further evaluated to see if this subgroup of trialists has insight into interim trial management practices that protect from trial bias. No demographic factor seemed to be associated with sharing the ACP at interim, which was corroborated with the sensitivity analysis. Due to some key limitations that include the high non-response and missing data, we caution interpreting the results as definitive, but rather they should be viewed as a first exploratory step to finding potential associations for further evaluation.

## Additional file

Additional file 1: Definitions of interim result measures. (PDF 22 kb)

## Abbreviations

ACP: Adaptive conditional power
DSMB: Data Safety Monitoring Board
HiREB: Hamilton Integrated Research Ethics Board
ICombinedER: Interim Combined Event Rate
IControlER: Interim Control Event Rate
ISCB: International Society of Clinical Biostatistics
MI: Multiple imputation
PI: Principle Investigator
SC: Steering Committee
SCT: Society of Clinical Trials
UCP: Unconditional conditional power
