# Supplementary material for: Exploring factors associated with views on sharing of certain interim trial result measures by the data safety monitoring board (DSMB) with non-DSMB members
Source: Trials. 2018 Nov 12;19:621. doi: 10.1186/s13063-018-2938-3 (PMC6233544; doi:10.1186/s13063-018-2938-3)
Supplement: Supplementary file 1 — Definitions of interim result measures. (PDF 22 kb) [file 13063_2018_2938_MOESM1_ESM.pdf]

Additional File 1: Definitions of interim results measures

Interim combined event rate (ICombinedER)	*“The total number of events observed at some planned interim point into the trial divided by the total number of participants admitted at that same planned interim point (e.g. a planned interim point can be six months from the start of the trial or after enrolling a certain number of participants).*<br><br>*Example:*<br><br>• *Total # of Deaths in both the placebo group and new intervention group, six months from the start of the trial = 80*<br>• *Total # of Participants in both the placebo group and the new intervention group, six months from the start of the trial = 700*<br>• *Calculation: 80/700 = 0.114 or 11.4%*<br>• *Therefore the Interim Combined Event Rate at the trial's interim analysis, six months from the start of the trial, is 11.4%”* [1]
Interim control event rate (IControlER)	The number of events observed among control participants at some planned interim point into the trial divided by number of control participants admitted at that same planned interim point (e.g. a planned interim point can be six months from the start of the trial)<br><br>Example:<br><br>• Total # of Deaths in the placebo group, six months from the start of the trial = 15<br>• Total # of Participants in the placebo group, six months from the start of the trial = 250<br>• Calculation: 15/250 = 0.06 or 6%<br>• Therefore the Interim Control Event Rate at the trial's interim analysis, six months from the start of the trial, is 6%
Adaptive conditional power (ACP)	*“The probability of rejecting the null hypothesis of no effect by the end of the trial (i.e. finding a statistically significant effect in favour of the intervention), at some predetermined interim point in the trial when the adaptive conditional power is scheduled to be calculated. The assumption made is that the observed interim effect (i.e. relative risk reduction) in the trial will remain the same till the end of the trial.*<br><br>*Example statement:*<br><br>*Given the interim data (data collected 2 years into the trial that is planned to last for 3 years), and assuming the observed interim effect (i.e. relative risk reduction) at the two year point to be the true effect for the remainder of the trial, the probability of rejecting the null hypothesis of no effect (i.e. finding a statistically significant effect in favour of the intervention) at the end of the trial is 60%.*<br>*The following pieces of information are used to calculate Adaptive Conditional Power at trial interim:*<br><br>• *Control event rate and experimental event rate*<br>• *Information Fraction; a ratio of the planned sample size and the number of patients recruited in trial at the interim analysis*<br>• *Z score and B value at interim*<br>• *Drift parameter”* [1]
Unconditional conditional power (UCP)	*“The probability of correctly rejecting the null hypothesis of no effect at the end of the trial (i.e. finding a statistically significant effect in favour of the intervention) and accepting the alternative hypothesis when indeed the alternative hypothesis is true, at some interim point in the trial.*<br><br>*The following pieces of information are used to calculate Unconditional Conditional Power at interim:*<br><br>*1. The hypothesized treatment effect at the design stage (i.e. relative risk reduction) of the trial, assuming the hypothesized treatment effect at the design stage to be true and correct for the remainder of the trial;*<br>*2. The sample size calculated at the design stage for the trial AND;*<br>*3. The combined event rate calculated at the trial's interim, assuming this rate to be true for the remainder of the trial.*<br><br>***Example statement:***<br>*Given the interim combined event rate and assuming the treatment effect (i.e. relative risk reduction) hypothesized at the design stage of the trial to be true for the remainder of the trial, the probability of correctly rejecting the null hypothesis of no effect (i.e. finding a statistically significant effect in favour of the intervention) at the end of the trial is 89%.”* [1]

[1] V. Borg Debono, L. Mbuagbaw, J. Paul, N. Buckley, L. Thabane, Sharing some interim data in trial monitoring can mislead or unmask trial investigators: A scenario-based survey of trial experts, Contemporary Clinical Trials Communications 7 (2017) 81-85.
